# Gut microbiota in Henoch-Schönlein purpura: from pathogenesis to therapeutic strategies

**DOI:** 10.3389/fimmu.2026.1838103

**Published:** 2026-05-21

**Authors:** Shuo Sun, Haiyan Lang, Sitong Cheng, Ruhua Ren, Wenjing Yao, Yucao Ma, Dalai Nashun, Yuhong Wang, Qin Si

**Affiliations:** 1 Department of Hematology, Dongzhimen Hospital, Beijing University of Chinese Medicine, Beijing, China; 2 Department of Hematology, International Mongolian Hospital of Inner Mongolia, Hohhot, Hohhot, Inner Mongolia Autonomous Region, China; 3 Innovative Mongolian Medical Engineering Research Centre, International Mongolian Hospital of Inner Mongolia, Hohhot, Inner Mongolia, Autonomous Region, China

**Keywords:** gut microbiota, Henoch-Schönlein purpura, immune dysregulation, intestinal barrier, pathogenesis, therapeutic strategies

## Abstract

Henoch–Schönlein purpura (HSP), also known as immunoglobulin A vasculitis, is a common systemic vasculitis in children. Although its pathogenesis remains unclear, recent studies suggest that the gut microbiota may play a significant role in its initiation and progression. In patients with HSP, gut microbiota dysbiosis and associated metabolic alterations are linked to impaired intestinal barrier integrity, activation of the innate immune system, and dysregulation of adaptive immune cell subsets; this includes imbalances in the T helper 17 (Th17)/regulatory T (Treg) and follicular helper T (Tfh)/follicular regulatory T (Tfr) axes. These changes may ultimately trigger immunoglobulin A immune complex deposition and dysregulation of the complement system, potentially establishing a positive feedback loop that drives immune-mediated inflammatory injury. Modulation of the gut microbiota has been shown to restore intestinal barrier function and immune homeostasis; this indicates its potential as a therapeutic target. This review summarizes recent research on gut microbiota alterations in patients with HSP, and evaluates its role in the pathogenesis of the condition. It also discusses promising therapeutic strategies, including probiotics and prebiotics, traditional Chinese medicine and its active components, fecal microbiota transplantation, and targeted-release formulations. This review aims to identify potential microbial biomarkers and therapeutic targets for improving the clinical management of HSP.

## 1 Introduction

Henoch–Schönlein purpura (HSP) is a form of non-thrombocytopenic purpura. It is pathologically characterized by inflammation in the walls of small blood vessels, consequent to the deposition of immunoglobulin A (IgA). Based on this pathological feature, the condition was reclassified as IgA vasculitis in 2012 (1). Clinically, it presents with palpable purpura and frequently involves multiple systems including the joints, gastrointestinal tract, and kidneys (2, 3). HSP more commonly affects children, showing a slight male predominance, and peaks in incidence during autumn and winter (4). Although the disease is often self-limiting, relapses are common. Renal involvement, which is observed in 20-80% of affected children, is a key determinant of long-term prognosis (5). Despite its lower incidence in adults, HSP carries a higher risk of severe renal impairment and poorer survival (6, 7). Environmental triggers, infections, and genetic predisposition are recognized etiological factors; however, the precise pathogenesis remains incompletely understood (2, 8). Current treatment of HSP is primarily symptomatic and supportive; glucocorticoids and other immunosuppressive agents are reserved for moderate to severe cases (3). However, prolonged use of these treatments carries considerable risks, including hypertension and increased susceptibility to infections (5).

**Figure 1:**
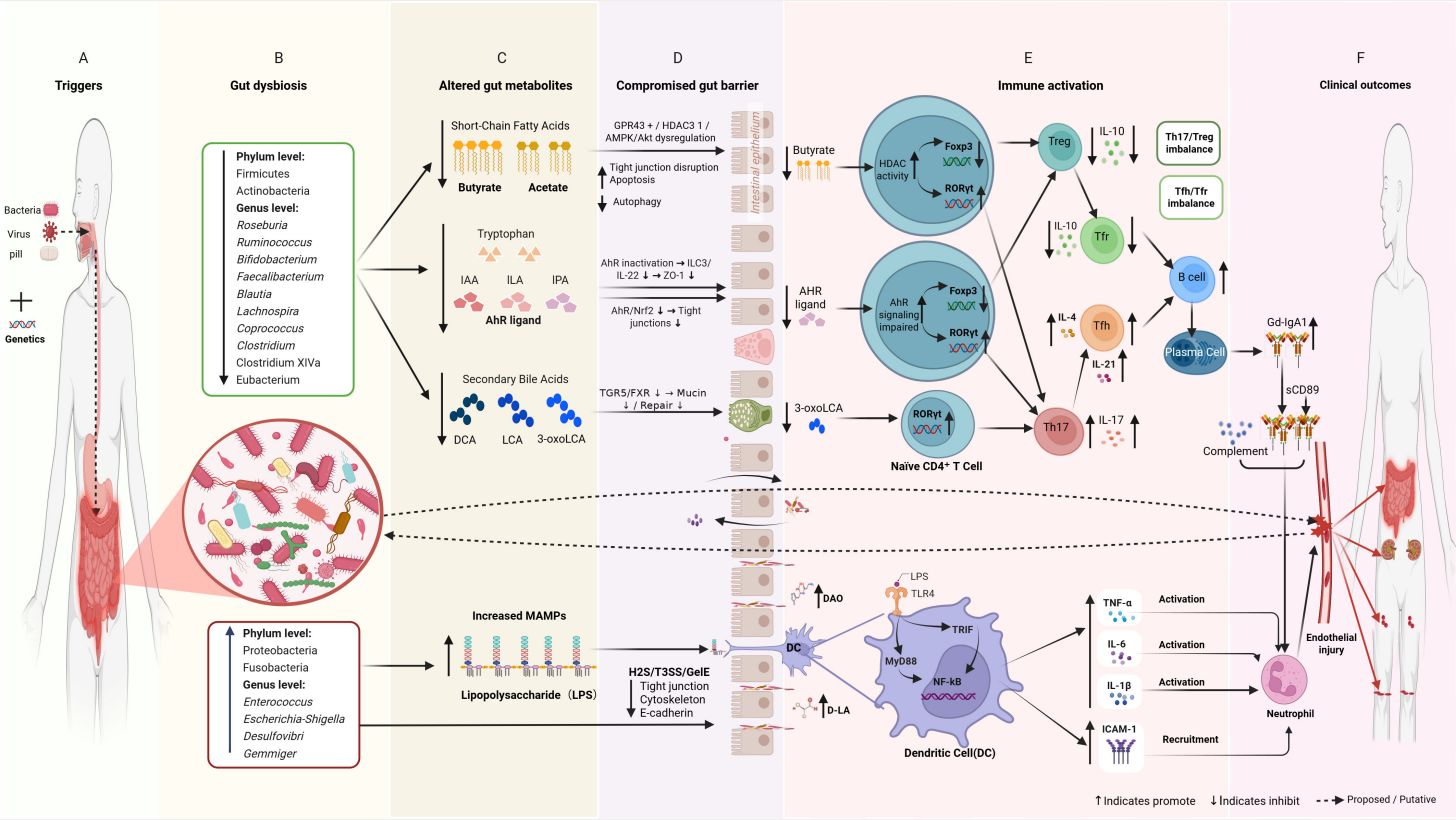
**(A)** Potential triggering factors (e.g., infection, genetic predisposition); **(B)** Gut microbiota dysbiosis, characterized by decreased abundance of SCFA-producing bacteria (e.g., Roseburia, Faecalibacterium) and increased abundance of opportunistic pathogens (e.g., Enterococcus); **(C)** Reduced levels of microbial metabolites (SCFAs, AhR ligands, lithocholic acid) and elevated levels of microbe-associated molecular patterns (e.g., LPS). **(D)** Intestinal barrier dysfunction, including reduced tight junction proteins and increased permeability; **(E)** Alterations in innate immunity (LPS/TLR4/NF-κB pathway) and adaptive immunity (Th17/Treg and Tfh/Tfr imbalances); **(F)** Production of pathogenic Gd-IgA1, immune complex deposition, vasculitis, and multi-organ involvement.

Recent evidence suggests that the gut microbiota-gut-immune axis may play a pivotal role in immune-mediated vasculitic diseases, attracting increasing attention (9–11). As the largest microbial community in the human body, the gut microbiota maintains a mutually beneficial relationship with the host and is essential for maintaining immune homeostasis, regulating inflammation, and preserving intestinal barrier function (12–14). Gut microbiota dysbiosis is being increasingly implicated as a key factor in the pathogenesis and progression of IgA-related diseases (9, 15). Its primary pathological mechanism may involve disruption of the microbiota-gut-immunity axis, leading to loss of homeostasis (10, 16). This disruption may drive disease progression through multiple mechanisms. First, dysbiotic microbiota may generate aberrant metabolites which stimulate intestinal lamina propria B cells and promote overproduction of pathogenic galactose−deficient IgA1 (Gd−IgA1) (15). Second, gut dysbiosis may reduce tight junction protein expression in intestinal epithelial cells, compromising barrier integrity and facilitating entry of microbial antigens into the bloodstream; this can promote aberrant systemic immune responses (17). Third, a reduction in symbionts that generate anti-inflammatory short-chain fatty acids (SCFAs), with a rise in proinflammatory bacteria, may disrupt cytokine balance via metabolic-immune interactions; this may impair immune tolerance (10). In conjunction, these interrelated pathways could contribute to the generation of pathogenic glycosylated IgA1 (Gd-IgA1), development of immune complexes, and consequent vascular inflammation (18) (**Figure 1**). Emerging evidence suggests that patients with HSP commonly demonstrate gut dysbiosis (9, 19–21), metabolic disturbances (22–24), impaired intestinal barrier function (25), abnormal cytokine profiles, and immune dysregulation (10, 26, 27). Specific alterations in the microbiota have also been found to correlate with disease activity (10, 21, 28, 29). This suggests that dysbiosis may be a key driver of disease pathogenesis rather than merely an accompanying phenomenon (9, 10, 15). Therefore, a comprehensive understanding of the mechanisms underlying the interplay between the gut microbiota and HSP is of theoretical and clinical value. It may clarify pathogenesis, identify novel therapeutic targets, and inform more precise treatment strategies (15, 29). This review therefore focuses on three key aspects: characterization of dysbiosis, mechanistic insights into pathogenesis, and emerging therapeutic strategies.

## 2 Gut microbiota dysbiosis and HSP

### 2.1 Gut microbiota composition in patients with HSP

The gut microbiota represents the most crucial and complex microbial ecosystem within the human body, where diverse bacterial species coexist and interact to collectively sustain its dynamic equilibrium (30). It is predominantly composed of several major phyla, including Firmicutes, Bacteroidetes, Proteobacteria, Actinobacteria, Verrucomicrobia, and Fusobacteria, with Firmicutes and Bacteroidetes usually dominating the community (31); certain Firmicutes species synthesize SCFAs (32). These metabolites serve as an energy source for intestinal cells and are crucial for preserving gut health (33). Based on their impact on human health, gut bacteria can be classified into three broad groups: (1) beneficial bacteria, such as probiotic genera *Bifidobacterium* and *Lactobacillus*, which are integral to the indigenous microbiota and contribute significantly to maintaining intestinal homeostasis (34), (2) opportunistic pathogens, including *Enterobacter* and *Enterococcus* species, which are typically harmless under normal conditions but may cause infection upon translocation to other sites (35), and (3) pathogenic bacteria, such as certain *Proteus* species, which are usually introduced exogenously or arise from abnormal overgrowth and can lead to intestinal infections or systemic inflammation (36).

High-throughput profiling techniques, particularly 16S rRNA and metagenomic sequencing, have been widely used to characterize the gut microbiome in HSP (37). Studies indicate that affected individuals demonstrate reduced alpha diversity, lower species richness, and impaired gut microbial defense functions compared to healthy controls (19, 38). This dysbiosis may contribute to the pathogenesis of HSP by impairing the intestinal barrier, inducing aberrant immune responses, and promoting systemic inflammation (9, 10, 27). Patients with HSP typically demonstrate reduced Firmicutes and increased Bacteroidetes in the gut microbiota, a pattern closely associated with chronic inflammation (16, 39). Simultaneously, beneficial bacteria with anti-inflammatory, immunomodulatory, and barrier-preserving functions (such as *Bifidobacterium* and *Blautia*) are significantly reduced (27), whereas potentially proinflammatory or opportunistic pathogens (such as *Fusobacterium* and *Enterococcus*) are relatively enriched (19, 21) (**Table 1**).

**Table 1:** Characteristics of gut microbiota in patients with Henoch-Schönlein purpura versus healthy controls.

Taxonomic level	Changes in HSP	Specific microbiota	Proposed mechanism	Underlying pathophysiological alterations	References
Phylum level	Decrease	Firmicutes	Butyrate production	Immune dysregulation; Barrier dysfunction; Reduced anti-inflammatory effect	(16, 19, 20, 27, 39)
		Actinobacteria	Mainly Bifidobacterium (see below)	Mainly Bifidobacterium (see below)	(29, 39)
	Increase	Proteobacteria	LPS source	Innate immune activation; Pro-inflammatory; Associated with HSP relapse; Associated with abdominal HSP (GI bleeding)	(16, 21, 28, 29)
		Fusobacteria	Not fully elucidated	Associated with abdominal HSP	(19, 21, 27)
		Verrucomicrobia	Mainly Akkermansia (see below)	Mainly Akkermansia (see below)	(27)
	Conflicting (mainly increase)	Bacteroidetes	Not fully elucidated	Increased in acute HSP; Decreased in relapsed HSP	(16, 21, 39)
Order level	Decrease	Lactobacillales	Restoration of microbiota balance; Promotion of mucin secretion; Promotion of sIgA secretion	Impaired intestinal barrier; Weakened immunity; Associated with renal HSP	(28)
Family level	Decrease	Lachnospiraceae	SCFA production	Th17/Treg imbalance; Positively correlated with IL-10	(9, 20)
		Bifidobacteriaceae	Resistance to pathogen colonization and invasion	Reduced intestinal epithelial barrier function; Associated with GI bleeding in HSP	(28)
		Ruminococcaceae	SCFA production	Th17/Treg imbalance, immune dysregulation	(9)
		Streptococcaceae	Not fully elucidated	Associated with GI bleeding in HSP	(28)
	Increase	Desulfovibrionaceae	Intestinal barrier damage	Associated with GI bleeding in HSP	(28)
		Barnesiellaceae	Pro-inflammatory	Impaired intestinal barrier function; Associated with renal HSP	(28)
Genus level	Decrease	Roseburia	Butyrate production	Impaired intestinal barrier; Immune dysregulation; Reduced anti-inflammatory effect; Negatively correlated with IgE level; Negatively correlated with hospital stay length	(9, 16, 19–21, 39)
		Ruminococcus	SCFA production (butyrate, propionate)	Immune dysregulation; Reduced anti-inflammatory effect; Impaired intestinal barrier; Associated with abdominal HSP	(9, 16, 20, 39)
		Bifidobacterium	Restoration of microbiota balance; Promotion of mucin secretion; Promotion of Paneth cell sIgA secretion	Reduced Treg, Th2 regulation; Immune dysregulation; Reduced inflammation suppression; Negatively correlated with renal HSP	(27, 28, 39)
		Faecalibacterium	SCFA production (butyrate); Anti-inflammatory peptide production	Reduced inflammatory regulation; Associated with abdominal HSP	(9, 20, 21)
		Blautia	Anti-inflammatory	Reduced inflammation suppression; Negatively correlated with TNF-α; Positively correlated with IL-10	(9, 27, 39)
		Lachnospira	SCFA production (butyrate, propionate); Promotion of microbiota balance	Impaired intestinal barrier; Immune dysregulation; Reduced anti-inflammatory effect; Reduced microbiota balance promotion	(9, 16, 20)
		Coprococcus	Butyrate production	Reduced anti-inflammatory effect; Negatively correlated with TNF-α; Associated with abdominal HSP	(9, 20)
		Clostridium	Butyrate production	Immune dysregulation; Associated with abdominal HSP	(9, 39)
		Clostridium XIVa	Butyrate production	Immune dysregulation	(27)
		Eubacterium	Butyrate production	Reduced anti-inflammatory effect	(20)
		Faecalibacterium prausnitzii	Butyrate production	Immune dysregulation; Reduced anti-inflammatory effect	(20)
	Increase	Enterococcus	Causes infection during dysbiosis	Negatively correlated with IgM; Shared maternal-child biomarker	(16, 19, 20)
		Escherichia-Shigella	Intestinal barrier damage; Causes infection during dysbiosis	Immune dysregulation; Closely associated with HSP relapse; Associated with renal HSP	(21, 41)
		Desulfovibrio	High H_2_S production; Cytochrome C oxidase inhibition; Intestinal epithelial cell damage	Impaired intestinal barrier function; Associated with GI bleeding; Associated with renal HSP	(28)
		Gemmiger	Impaired microbiota balance; Promotion of inflammation	Chronic intestinal inflammation; Positively correlated with IL-6, IgA, IgG; Associated with renal injury in HSP	(28)
		Veillonella	Not fully elucidated	Associated with GI tract involvement in acute phase	(39)
	Conflicting (mainly increase)	Parabacteroides	Downregulation of occludin and ZO-1	Impaired intestinal barrier; Positively correlated with IgE level; Associated with GI bleeding	(19, 21, 28, 39)
		Bacteroides	SCFA production; Causes infection during dysbiosis	Affects B cell metabolism; Affects IgA and IgG levels; Positively correlated with IgG; Associated with renal HSP	(21, 39, 41)
		Prevotella	LPS source	Immune dysregulation; Pro-inflammatory; Positively correlated with IL-8; Associated with renal HSP	(27, 28, 39)
	Conflicting (mainly decrease)	Dialister	Anti-inflammatory	Negatively correlated with eczema	(16, 19, 21)
	Conflicting	Streptococcus	Causes infection during dysbiosis; Macrophage activation (TLR pathway); Surface antigens form immune complexes with IgA1	Immune dysregulation; Pro-inflammatory; Associated with abdominal HSP; Positively correlated with renal HSP severity; Associated with hematuria	(16, 20, 21, 41)
		Fusobacterium	Butyrate production	Increased in abdominal HSP; Decreased in cutaneous HSP	(21, 28)
		Akkermansia	Bidirectional regulation of barrier function	Affects intestinal barrier function and immune regulation	(9, 27)
Species level	Decrease	Blautia wexlerae	SCFA production (mainly butyrate)	Impaired Treg differentiation, immune dysregulation; Reduced colonic mucus maintenance, impaired barrier function; Reduced anti-inflammatory effect; Positively correlated with IL-10	(27)
		Bifidobacterium pseudolongum	Inhibition of pro-inflammatory cytokines	Reduced Treg/Th2 regulation; Reduced gut microbiota improvement function	(27)
		Bifidobacterium pseudocatenulatum	Metabolic promotion; Pathogen inhibition	Reduced intestinal defense capacity	(20)
		Bacteroides fragilis	Polysaccharide A source	Th1/Th2 imbalance; Reduced Treg function	(27)
	Increase	Akkermansia muciniphila	Mucin degradation	Impaired barrier function; Positively correlated with IL-6, IL-12, IL-17	(27)
		Prevotella copri	Pro-inflammatory	Induces Th17-mediated immune response and promotes IL-17 production	(27)

### 2.2 Gut microbiota signatures across different clinical phenotypes

Compared with healthy children, those with cutaneous HSP demonstrate reduced abundance of the phylum Actinobacteria, significantly decreased levels of the genera *Fusobacterium* and *Bifidobacterium*, and lower alpha diversity (28, 29). Abdominal-type HSP is associated with increased abundance of the Proteobacteria phylum, significantly increased levels of the genera *Streptococcus* and *Fusobacterium*, and reduced *Faecalibacterium*; these patients show higher α-diversity than those with non-abdominal phenotypes (21, 29, 40). In the gastrointestinal bleeding phenotype, the levels of Proteobacteria and Desulfovibrionaceae are significantly increased, whereas those of Bifidobacteriaceae and Streptococcaceae are markedly decreased (28). In renal HSP (HSPN), the levels of *Bacteroides*, *Escherichia–Shigella*, *Streptococcus*, *Prevotella*, and *Desulfovibrio* are significantly increased, whereas levels of Lactobacillales and *Bifidobacterium* are significantly decreased (28, 41). Notably, increased abundance of *Streptococcus* correlates with the severity of renal involvement (40). Higher levels of *Streptococcus* are associated with an increased likelihood of hematuria, hypoalbuminemia, and reduced microbial diversity and richness (40, 41).

Gut microbiota alterations have been associated with the severity of organ damage and clinical manifestations in HSP. Liu et al. reported that the levels of *Bifidobacterium* and *Lactobacillus* gradually decrease from healthy controls to those with HSP and further to those with HSPN, whereas levels of *Escherichia coli* and *Streptococcus* gradually increase. In addition, dysbiosis is more pronounced in children with severe HSPN than in those with mild disease (42). Another study found that organ involvement was associated with elevated Proteobacteria and reduced Actinobacteria, suggesting a potential role for these phyla in the systemic manifestations of HSP (29). Abdominal symptoms of HSP are associated with *Streptococcus* and butyrate-producing bacteria. HSP recurrence is associated with increased Proteobacteria and *Escherichia–Shigella*, which is markedly enriched in the recurrent stage (21, 40). Correlation analyzes have revealed positive association of *Bacteroides* with serum IgG levels and *Prevotella* with serum IL-8 levels, and negative association of *Bifidobacterium* with serum IL-10 levels and *Lachnospiraceae* with complement C3 levels (21, 28).

These findings suggest that gut microbiota profiling facilitates risk stratification in patients with HSP and enables early identification of those at high risk for severe complications, including renal involvement and disease relapse (21, 28, 42).

### 2.3 Dynamic changes between acute and remission phases

Although available evidence is predominantly derived from cross-sectional studies, several investigations have directly compared gut microbiota characteristics between the acute and remission phases in pediatric HSP patients.

Compared with healthy children, those with active HSP show dysregulated gut microbiota composition. Liang et al. reported significantly reduced gene copy numbers of *Bifidobacterium* and *Lactobacillus* in affected infants, along with an immune imbalance characterized by decreased Treg cells and increased Th17 cells (43). Using metagenomic approaches, Cao et al. further demonstrated that the gut microbiota in treatment-naïve patients with acute-stage HSP is dominated by Gram-positive bacteria, and shows deviations in both taxonomic composition and functional pathways from the normal baseline (20). In the gastrointestinal tract involvement subtype, Zhang et al. used high-throughput sequencing to analyze fecal samples from pediatric patients; they found a significantly higher relative abundance of *Veillonella* during the acute phase than in the recovery phase (39).

After clinical remission, certain beneficial microbial taxa show signs of recovery. Liang et al. reported increased copy numbers of *Bifidobacterium* and *Lactobacillus* during remission in pediatric patients, primarily driven by *Bifidobacterium*. However, the Treg/Th17 imbalance remained uncorrected, suggesting that gut dysbiosis and immune dysregulation may persist as pathological alterations independent of clinical symptoms (43). Using longitudinal data, Cao et al. further demonstrated that although certain beneficial taxa such as *Faecalibacterium prausnitzii* and *Bifidobacterium pseudocatenulatum* increased during remission, the overall microbiome profile did not fully return to the state seen in healthy controls (20). In pediatric patients with abdominal-type HSP, Zhang et al. found *Ruminococcus* to be most abundant during the convalescent stage (39).

## 3 Role of gut microbiota dysbiosis in the pathogenesis of HSP

Emerging evidence on the association between gut microbiota and HSP, together with preliminary causal evidence from fecal microbiota transplantation (FMT), suggests that gut dysbiosis may contribute to the initiation and progression of HSP. This may occur via multiple interconnected pathways, including the modulation of microbial metabolites, disruption of intestinal barrier function, activation of innate immunity, and promotion of adaptive immune dysregulation (10, 19, 27). Conversely, the systemic inflammatory state in HSP may exacerbate gut dysbiosis and impair barrier function, potentially establishing a bidirectional vicious cycle (15, 44).

### 3.1 Gut microbiota metabolite disturbances in HSP

The gut microbiota converts dietary components and host-derived substances into a variety of bioactive molecules through metabolic activity; SCFAs, tryptophan metabolites, and secondary bile acids (SBAs) are the major classes (45). In patients with HSP, these alterations in gut microbial composition are associated with corresponding changes in these metabolites (22–24, 27). However, it remains unclear whether these metabolic alterations are primary drivers of disease or secondary consequences of systemic inflammation. This section therefore summarizes the metabolic changes observed in HSP; their mechanistic contributions to barrier dysfunction and immune regulation are discussed in Sections 3.2 and 3.4, respectively.

#### 3.1.1 Reduction of SCFAs

SCFAs, mainly acetate, propionate, and butyrate, are important metabolic products produced by intestinal microbes via anaerobic fermentation of dietary fiber (32, 46). These fatty acids are predominantly synthesized by specific genera within the Firmicutes and Actinobacteria phyla. Butyrate is mainly generated by *Faecalibacterium*, *Lachnospira*, and *Roseburia* (Firmicutes), whereas acetate is primarily produced by *Bifidobacterium* (Actinobacteria) and certain *Blautia* (Firmicutes) species, which also contribute to butyrate production (32, 33). Additionally, *Bifidobacterium* and *Clostridium* cluster XIVa contribute to butyrogenesis indirectly via metabolic cross-feeding interactions (47, 48). In patients with HSP, reduced abundance of these bacteria may result in decreased intestinal SCFA levels, particularly butyrate and acetate (9, 27, 39). This is supported by clinical fecal analyzes, which show marked reduction of fecal acetate and butyrate in the acute phase and partial recovery (often remaining subnormal) of acetate during convalescence; however, butyrate remains persistently low (22).

#### 3.1.2 Impaired tryptophan metabolism

Homeostasis of tryptophan, an indispensable amino acid derived exclusively from dietary sources, is regulated by the gut microbiota (49). This regulation occurs via two competing metabolic pathways, namely, the synthesis of indole-derived compounds and the enzymatic kynurenine pathway (50). In patients with HSP, reduced levels of beneficial bacteria, particularly within the Firmicutes and Actinobacteria phyla, are associated with decreased synthesis of indole-derived metabolites, which normally function as ligands for the aryl hydrocarbon receptor (AhR). This may impair the protective indole-AhR pathway, although causality requires further mechanistic investigation (51). Research indicates that specific gut bacteria, including *Lactobacillus*, *Bifidobacterium pseudolongum, and Bacteroides fragilis*, facilitate the enzymatic metabolism of tryptophan (52). This process, mediated by tryptophanase, yields indole-based metabolites including indole-3-acetic acid and indole-3-lactic acid (51, 52). Clinical hematological analyzes support this association, as plasma tryptophan levels are reduced in children with acute-phase HSP (24).

#### 3.1.3 Decreased SBAs

SBAs, including deoxycholic and lithocholic acids, are produced from primary bile acids via 7α-dehydroxylation. This biochemical conversion is mediated by strictly anaerobic gut bacterial species (53), which primarily include 7α-dehydroxylating members of the Firmicutes phylum, clusters from *Clostridium* XIVa, and members of *Ruminococcus* (54). In patients with HSP, a marked reduction in these key bacterial populations may contribute to reduced SBA synthesis (10, 16, 23). In addition, the expansion in potential pathogenic bacteria such as Escherichia–Shigella and *Desulfovibrio* may induce intestinal inflammation and oxidative stress, thereby creating unfavorable conditions for strictly anaerobic 7α-dehydroxylating bacteria; in turn, this could contribute to reduced synthesis of SBAs (21, 28). The reduction in SBAs, which possess intrinsic antimicrobial activity (55), may also promote the expansion of opportunistic pathogens, thereby reinforcing a self-perpetuating cycle that exacerbates intestinal dysbiosis (56). This aligns with clinical evidence, which indicates that in children with acute HSP, fecal SBAs (deoxycholic and lithocholic acids) are significantly reduced relative to healthy controls, while primary bile acids (cholic and chenodeoxycholic acids) are elevated during convalescence (23).

### 3.2 Gut barrier dysfunction in HSP

Gut microbiota dysbiosis has been linked to the onset of vascular inflammation in HSP, potentially via disruption of intestinal barrier integrity (19, 25). Under healthy conditions, the commensal microbiota play an important role in maintaining barrier integrity. In contrast, dysbiosis is associated with metabolic disruption and a loss of protective function, which may increase intestinal permeability (57, 58). The subsequent translocation of antigens and microbial components is associated with sustained systemic immune activation and may contribute to the progression of HSP pathology (14).

#### 3.2.1 Microbial dysbiosis and barrier integrity

Patients with HSP exhibit an increased relative abundance of Desulfovibrio (28), *Enterococcus* (19), and *Escherichia–Shigella* (21). The genus *Desulfovibrio*, comprising sulfate-reducing bacteria, generates hydrogen sulfide (59). Increased concentrations of this metabolite have been shown to cause cellular toxicity by suppressing the synthesis of proteins essential for tight junction integrity, thereby compromising intestinal barrier function (60). Recent studies suggest that *Escherichia–Shigella* uses a type III secretion apparatus to translocate effector proteins into host cells (61). This process appears to trigger actin cytoskeleton reorganization, facilitate intercellular bacterial spread, and promote epithelial cell death and barrier dysfunction in experimental models (62–64). Escherichia–Shigella further facilitates host cell entry by co-opting human intestinal defensin 5 through interaction with P2Y11 purinergic receptors on colonic epithelial cells. This binding stimulates a signaling cascade involving cyclic adenosine monophosphate (AMP) and subsequent activation of protein kinase A. Ultimately, this triggers filopodia formation, thereby enhancing bacterial internalization (61). Certain opportunistic enterococci, including *Enterococcus faecalis*, produce the metalloprotease GelE which breaks down E-cadherin and thereby weakens the colonic epithelial barrier. This increases intestinal permeability, allowing gut microbiota, toxins, and various antigens to cross the barrier more readily. The process subsequently activates immune responses and may initiate or exacerbate chronic inflammatory conditions (65).

#### 3.2.2 Metabolite alterations and barrier function

As described in Section 3.1.1, patients with HSP show a reduced abundance of SCFA-producing bacteria, including those responsible for butyrate production, along with decreased butyrate levels. Butyrate serves as the primary metabolic substrate for the energy requirements of colonocytes (66). In addition, it can enhance intestinal mucosal barrier function via multiple mechanisms. It can activate G protein-coupled receptor 43 and inhibit histone deacetylase 3, thereby regulating the AMP-activated protein kinase (AMPK)/protein kinase B (Akt) signaling pathway. This downstream signaling inhibits intestinal epithelial cell apoptosis and promotes autophagy, thereby contributing to barrier maintenance (67, 68). Butyrate primarily exerts barrier-protective effects by reducing intestinal permeability. This is mediated via specific mechanisms including restoration of tight junction integrity (zonula occludens-1, claudin, and occludin), upregulation of mucin 2 and antimicrobial peptide expression, and maintenance of the mucus barrier via secretory IgA and goblet cells. Butyrate supplementation has been shown to restore compromised intestinal barrier function (68, 69). In addition, recent studies show that Bifidobacterium can enhance the intestinal tight junction barrier by upregulating occludin gene expression via the Toll-like receptor 2 and Toll-like receptor 6 complex (70).

Disruption of tryptophan metabolism also compromises barrier integrity. As noted in Section 3.1.2, patients with HSP exhibit reduced production of AhR-activating indole derivatives (24, 49). This leads to insufficient activation of the AhR pathway; in turn, this impairs interleukin-22 secretion by group 3 innate lymphoid cells residing in the intestinal lamina propria (71). Interleukin-22 plays a key role in gut health by promoting epithelial repair and antimicrobial peptide production; this supports mucosal barrier restoration and strengthens antimicrobial defense (72, 73). AhR signaling also plays a vital role in sustaining the functional integrity of intestinal intraepithelial lymphocytes and goblet cells (74). Activation of AhR upregulates zonula occludens-1 expression in the intestinal epithelium, leading to decreased barrier permeability (75, 76). A disrupted tryptophan-AhR axis therefore weakens both chemical and immunological barriers of the gut (52). This disruption facilitates pathogen and antigen translocation into the circulation, thereby exacerbating systemic immune dysregulation and inflammation (77). Tryptophan metabolism disorders also involve AhR-nuclear factor erythroid 2-related factor 2-mediated regulation of tight junction proteins and kynurenine pathway dysregulation leading to epithelial apoptosis (76, 78, 79).

In addition to butyrate and tryptophan metabolites, SBAs play a critical role in maintaining epithelial barrier function. As described in Section 3.1.3, SBA levels are reduced in HSP (23). Under physiological conditions, SBAs stimulate goblet cell mucin production and promote epithelial cell migration and renewal, thereby strengthening the mucosal barrier (80–82). They also act as endogenous ligands for Takeda G protein-coupled receptor 5 and modulate farnesoid X receptor signaling, thereby suppressing bacterial overgrowth and maintaining epithelial integrity (81, 83). A decline in SBAs may lead to insufficient farnesoid X receptor/Takeda G protein-coupled receptor 5 activation; this may weaken barrier function and create a microenvironment conducive to pathogenic bacterial colonization (84, 85).

### 3.3 Innate immune activation in HSP

Accumulating evidence suggests that gut dysbiosis may contribute to the pathogenesis of HSP by activating innate immune pathways. In patients with HSP, alterations in the gut microbiota may compromise intestinal barrier integrity and increase its permeability (19, 25). Such pathological changes may promote the translocation of microorganism-associated molecular patterns (MAMPs) (86). These molecules may be recognized by innate immune cells via pattern recognition receptors, leading to activation of the nuclear factor kappa-B (NF-κB) signaling pathway (87). Activation of this pathway may increase the secretion of proinflammatory cytokines, including TNF-α, IL-1β, and IL-6, thereby sustaining a persistent inflammatory state (88, 89). This ongoing inflammation may further exacerbate microbial dysbiosis and compromise barrier integrity (10, 27), ultimately establishing a self-perpetuating cycle that contributes to the onset and progression of HSP (9, 19, 26).

#### 3.3.1 Increased intestinal permeability

Substantial evidence supports the presence of intestinal barrier dysfunction in patients with HSP, as reflected by markedly elevated serum levels of diamine oxidase, D-lactate (D-LA), and endotoxin. These changes are consistent with the hypothesis of increased intestinal permeability (25). Elevated serum levels of diamine oxidase, an enzyme located in intestinal mucosal epithelial cells, are widely regarded as an indirect marker of impaired mucosal barrier integrity (90). Similarly, increased D-LA, primarily derived from gut bacteria, suggests translocation of bacterial products through mucosal defects (25). Translocation of endotoxin (lipopolysaccharide [LPS]) further indicates reduced ability of the intestinal barrier to restrict microbial components (91). Although increased intestinal permeability has been proposed to facilitate translocation of MAMPs and trigger systemic immune activation (17, 25), this immune response may further impair intestinal barrier dysfunction, thereby establishing a vicious cycle.

#### 3.3.2 MAMP translocation and innate immune activation

Accumulating evidence suggests that increased intestinal permeability may facilitate the translocation of MAMPs into the submucosa and systemic circulation; this is recognized as a key mechanism for triggering innate immune responses (17, 92). Among these MAMPs, LPS is a core structural component of the outer membrane of Gram-negative bacteria, particularly Proteobacteria. Studies in experimental models have shown that LPS may potentially disrupt intestinal barrier integrity and activate innate immunity (20, 92, 93).

Existing studies indicate that LPS primarily induces inflammation by activating the Toll-like receptor 4 (TLR4) signaling pathway. It is recognized by the TLR4/myeloid differentiation factor 2 complex on the surface of immune cells and vascular endothelial cells; this subsequently activates the myeloid differentiation primary response protein 88 (MyD88)-dependent cascade. This cascade involves sequential activation of the interleukin-1 receptor-associated kinase family, TNF receptor-associated factor 6, and the IκB kinase complex, ultimately promoting IκB phosphorylation and degradation. This allows NF-κB to translocate into the nucleus and initiate the transcription of proinflammatory cytokines (such as TNF-α, IL-1β, and IL-6) (92–94). TLR4 can also initiate TRIF-dependent non-MyD88-mediated signaling, which is finely regulated by cell type, microenvironment, and various negative feedback regulators including Suppressor of Cytokine Signaling 1 (95, 96).

Evidence suggests that upon TLR4 activation, downstream inflammatory mediators may compromise intestinal barrier integrity through multiple mechanisms. In cellular or animal models, activated myosin light chain kinase has been found to induce phosphorylation of tight junction proteins, thereby impairing tight junction function (14, 70). In parallel, NF-κB-mediated signaling promotes the production of TNF-α and other cytokines, which upregulate claudin-2 (which forms paracellular “leaky” channels) while downregulating barrier-forming proteins such as claudin-1 and occludin (14, 97). Collectively, these alterations support the hypothesis that LPS-induced TLR4-driven inflammation may establish a self-perpetuating cycle, which promotes metabolic dysregulation and further increases intestinal permeability (81, 82). In addition, circulating levels of LPS have been demonstrated to correlate positively with the relative abundance of Proteobacteria, suggesting that gut microbiota dysbiosis may amplify this proinflammatory cascade (14).

In addition to LPS, other MAMPs also contribute to innate immune activation. For instance, certain pro-inflammatory species within the genus *Prevotella* (such as *Prevotella copri*, which has been reported to be enriched in HSP) are known to engage Toll-like receptor 2 and influence mucosal immune responses (98, 99). In contrast, specific strains of *Bifidobacterium* enhance intestinal barrier function by inducing antimicrobial peptide REGIIIγ production via MyD88-dependent Toll-like receptor signaling (100). In agreement with these findings, patients with HSP exhibit gut dysbiosis, which is characterized by a reduction in protective genera such as *Bifidobacterium* and expansion of *Prevotella* (27). Dysregulated Toll-like receptor signaling, through either aberrant activation or impaired homeostasis, can drive immune imbalance and contribute to the pathogenesis of various inflammatory, autoimmune, and neoplastic diseases (92, 101).

#### 3.3.3 Immune activation and vascular injury

A current hypothesis suggests that translocation of microbial components, particularly LPS, triggers systemic innate immune activation. This may exacerbate local intestinal injury and contribute to the development of vascular lesions characteristic of HSP (92, 102). A central tenet of this hypothesis is supported by preclinical studies, which show that upon entering the bloodstream, bacterial components such as LPS can activate vascular endothelial cells *in vitro* and in animal models (93, 103). This activation is believed to facilitate the deposition of IgA1-containing immune complexes within the vessel wall, a defining feature of HSP (2, 26). *In vitro* studies have demonstrated that LPS upregulates the expression of endothelial cell adhesion molecules (such as ICAM-1) via the TLR4/NF-κB pathway (93, 104), thereby promoting transendothelial migration of neutrophils (105). Although this sequence exacerbates vascular inflammation in experimental models and has been proposed to contribute to the pathogenesis of HSP, its precise order of occurrence, predominant role, and clinical relevance in patients require further validation (26).

### 3.4 Dysregulation of adaptive immunity in HSP

The immunopathogenesis of HSP is associated with aberrant adaptive immune responses, primarily characterized by an imbalance between proinflammatory Th17 lymphocytes and immunosuppressive Tregs, along with disruption of Tfh/Tfr equilibrium (27, 106). The gut serves as a central hub for immune regulation, where microbial homeostasis sustains systemic immune tolerance through bacterial metabolites and direct intercellular interactions (106–108). Gut microbiota dysbiosis may disrupt the development and function of Th17, Treg, Tfh, and Tfr cells through multiple molecular pathways (109–111), thereby exacerbating vascular endothelial inflammation (26).

#### 3.4.1 Gut microbiota dysbiosis and Th17/Treg imbalance

A reduction in protective commensal bacteria may impair the maintenance of immune tolerance (12, 35). For instance, an abundance of the genus *Blautia* has been found to correlate positively with intestinal interleukin 10 (IL-10) levels in patients with HSP; this suggests that its depletion may reduce Treg differentiation and IL-10 secretion, thereby partially compromising immunoregulatory function (27, 39). In parallel, overgrowth of opportunistic pathogens may exacerbate the inflammatory response (35, 36). In patients with HSP, increased abundance of Proteobacteria correlates with elevated levels of LPS. *In vitro*, LPS activates dendritic cells via the MyD88-dependent TLR4 pathway and promotes IL-23 production (16, 92); this cascade may contribute to Th17 cell expansion and exacerbate inflammatory responses (57, 109). Collectively, gut microbiota alterations may shift the immune balance toward a proinflammatory state by suppressing Treg function and enhancing Th17 responses (27, 109, 112).

#### 3.4.2 Dysregulation of metabolites and imbalance of the Th17/Treg axis

Based on the metabolic alterations described in Section 3.1, levels of three categories of microbiota-derived metabolites (SCFAs such as butyrate, tryptophan-derived AhR ligands, and SBAs) are reduced. These coordinated metabolic changes may collectively regulate the Th17/Treg balance, a central determinant of immune homeostasis in HSP (113–115).

Evidence suggests that butyrate may promote Treg differentiation and inhibit Th17 polarization. *In vitro* and animal studies suggest that butyrate acts as a histone deacetylase (HDAC) inhibitor. This activity increases acetylation at the forkhead box P3 (Foxp3) locus, thereby upregulating expression of this key transcription factor for Treg (116, 117). Observational studies in patients with HSP during the acute phase show that reduced fecal butyrate levels are associated with decreased numbers of peripheral blood Tregs, downregulated Foxp3 expression, and impaired Treg suppressive function (22, 118). Low butyrate levels may also be associated with increased Th17 differentiation, as butyrate−mediated inhibition of HDAC can reduce the expression of retinoic acid receptor−related orphan receptor gamma t (RORγt) (104).

Experimental studies indicate that tryptophan−derived ligands for the AhR can regulate CD4^+^ T cell differentiation. Among these, kynurenine, generated via the indoleamine 2,3−dioxygenase pathway, acts as a high−affinity endogenous ligand (75, 119). Under *in vitro* conditions in the presence of transforming growth factor-β (TGF-β), kynurenine activates the AhR. This activation promotes Foxp3 expression, favors the differentiation of naive CD4^+^ T cells toward the Treg lineage, and inhibits RORγt mediated Th17 differentiation (110, 120). Patients with HSP in the acute phase show decreased indoleamine 2,3−dioxygenase activity and reduced kynurenine levels, along with impaired AhR signaling (24, 50). Based on the available mechanistic evidence, this defect may shift the Treg/Th17 balance toward Th17 polarization (71, 121).

*In vitro* studies have shown that 3 oxolithocholic acid, a SBA metabolite, directly binds to RORγt; this interaction inhibits its transcriptional activity, leading to reduced IL17 levels and suppressed Th17 cell differentiation (122). In patients with HSP, dysbiosis is associated with reduced levels of SBAs. This may attenuate RORγt inhibition, thereby favoring Th17 cell expansion (23, 123, 124). Additionally, isolithocholic acid has been reported to promote Treg expansion and upregulate Foxp3 expression by inducing mitochondrial oxidative stress (123). Animal studies have shown that supplementation with these metabolites alters the distribution of intestinal T cell subsets, decreasing Th17 cells and increasing Treg cells (122, 123).

In summary, reduced levels of these three classes of metabolites, namely butyrate, AhR ligands, and 3 oxolithocholic acid, are associated with a shift of the Th17/Treg balance toward a proinflammatory phenotype (109, 125, 126). Each metabolite can either enhance Treg or inhibit Th17 through distinct but complementary mechanisms *in vitro* or in animal models (127, 128). Based on these findings, we hypothesize that the concurrent reduction of these metabolites in patients with HSP may synergistically contribute to immune dysregulation, thereby impairing self−tolerance and promoting vasculitis (22–24). This convergence indicates a complex interaction among the gut microbiota, its metabolites, and immune polarization (10, 27, 129).

However, most of the studies cited in this section are cross−sectional in design. Although they suggest that microbial metabolites may influence the Th17/Treg balance, reverse causality cannot be excluded, as HSP-associated inflammation may itself drive alterations in metabolite profiles. Longitudinal or interventional studies are therefore needed to validate these findings.

#### 3.4.3 HSP and Th17/Treg imbalance

In patients with HSP, elevated serum levels of interleukin-17 and interleukin-4, along with reduced concentrations of interferon-γ and IL-10, may suggest aberrantly enhanced Th17 and T helper 2 (Th2) responses, accompanied by impaired Treg-mediated immunosuppression (27). Single−cell sequencing and flow cytometry analysis provide further evidence at the level of circulating immune cells, showing that patients with HSP exhibit increased populations of effector/memory T (including CD8^+^ T, Tfh, Th2, and Th17), myeloid, and humoral immune (plasma and B) cells, along with decreased levels of naïve CD4^+^ T, regulatory Treg, T helper 1, and natural killer cells (130). The study further indicates that these expansion patterns exhibit clinical subtype heterogeneity; cytotoxic effector T cells predominate in the cutaneous/renal subtype, whereas plasma, B, and Tfh cells are more common in the articular/abdominal subtype.

Recent FMT studies provide preliminary experimental support for a potential causal relationship between gut dysbiosis and HSP pathogenesis. Chen et al. transplanted gut microbiota from pediatric patients with HSP into recipient mice and observed significant alterations in the gut microbiota composition, accompanied by elevated levels of IL−17A, interleukin−21, and IgA (10). These findings suggest that gut dysbiosis may contribute to the characteristic inflammatory and immune responses in HSP; however, further validation is required in larger cohorts. Overall, the available evidence supports an association between gut dysbiosis and autoimmune inflammation characterized by vascular IgA deposition in HSP (8, 27).

#### 3.4.4 HSP and Tfh/Tfr imbalance

In addition to the Th17/Treg axis, the balance between Tfh and Tfr cells has been proposed as a key regulatory mechanism in humoral immunity, particularly in the control of IgA production. Tfh cells, characterized by the expression of C-X-C motif chemokine receptor 5 and B cell lymphoma 6 (Bcl-6), facilitate B cell differentiation and immunoglobulin class-switch recombination within germinal centers. Conversely, Tfr cells, which co-express Foxp3 and Bcl-6, suppress excessive antibody responses (131–133). Disruption of the Tfh/Tfr balance has been implicated in various IgA-associated disorders, including IgA nephropathy and HSP (106, 134).

Mechanistically, Th17 and Tfh cells share common regulatory pathways, such as the PI3K-mTOR axis. Under pathological conditions like Roquin deficiency, concurrent elevations of Th17 and Tfh cells are observed. Meanwhile, because Tfr cells originate from Treg cells, the quantity and functional status of Treg cells directly determine the reservoir of Tfr cells. This suggests a close correlation between Th17/Treg imbalance and Tfh/Tfr imbalance (135).

Studies indicate that in patients with HSP, particularly those with renal involvement, the Tfh/Tfr ratio is elevated; this is driven by expansion of Tfh cells and concomitant reduction in Tfr cells (106). This imbalance is accompanied by increased Bcl-6 mRNA expression and decreased Blimp-1 mRNA expression in CD4+ T cells, along with elevated levels of IL-21 and IL-6 (106, 134). Compared to healthy controls, children with HSP exhibit significantly increased circulating total B cell counts. This is accompanied by a reduction in the proportions of plasma and naïve B cells, and expansion of memory unswitched B cells. Notably, the increased frequency of Tfh cells correlate significantly with these alterations in B-cell subsets (136). In addition, serum levels of IgA1 and Gd-IgA1 are significantly elevated in patients with HSP, especially in those with HSPN (134). Bioinformatics analysis of transcriptomic data from patients with HSPN indicates that hyperdifferentiation of Th2, Th17, and Tfh cells, as well as activation of TLR signaling pathways, may play important roles in the onset and progression of the disease (111). Collectively, these findings suggest that Tfh/Tfr imbalance may contribute to the pathogenesis of HSP by promoting excessive B cell activation and the production of aberrantly glycosylated IgA1 (106, 134). Emerging evidence indicates that gut microbiota-derived metabolites can regulate the differentiation and function of Tfh cells. For instance, dysbiosis of butyrate-metabolizing bacteria has been shown to promote Tfh cell differentiation while suppressing regulatory Treg cells (137, 138). Consistently, evidence from Tfr-deficient mouse models has confirmed that Tfr cells maintain gut microbiota homeostasis by regulating the production of microbiota-specific IgA (131). It is reasonable to propose that intestinal dysbiosis in HSP contributes not only to Th17/Treg imbalance, but also to Tfh/Tfr dysregulation. Future studies are warranted to investigate whether microbiota−targeted interventions can restore Tfh/Tfr balance and reduce pathogenic IgA production (111).

### 3.5 Aberrant IgA response and vascular injury in HSP

Available evidence suggests that the onset and progression of HSP are driven by a multifactorial process, in which abnormally Gd-IgA1 and its recognition by antiglycan antibodies play a central role in most proposed models (26, 139). However, the predominant mechanisms may vary among patients. In the context of intestinal homeostasis, secretory IgA operates in a polyreactive and immunologically tolerant “sensing, regulating, and tolerating” mode to maintain microbial commensalism (140, 141). Gut microbiota dysbiosis is believed to disrupt this balance, promoting a shift in the mucosal immune microenvironment from a regulatory phenotype (Treg/Tfr predominance) toward an inflammatory phenotype (Th17/Tfh predominance) (109, 116, 118). This transition has been associated, in animal models and some human studies, with a shift in IgA function from a protective to a pathogenic one (26). Translocated gut microbial antigens may activate dendritic cells. In turn, these cells may aberrantly activate B cells via T cell-dependent or -independent pathways and molecular mimicry mechanisms, thereby promoting class switching to IgA (141–144). However, the relative contributions of these pathways in humans remain incompletely understood. Microbial signals are sensed via the TLR pathway and may be amplified by cytokines such as interleukin−21 produced by Tfh cells, collectively promoting B cell activation and IgA class switch recombination (2, 8, 144, 145). Dysregulation of inflammatory cytokines may affect the expression and activity of core 1 β1,3-galactosyltransferase, resulting in impaired O-glycosylation of the IgA1 hinge region and increased production of Gd-IgA1 (146). However, other genetic or environmental modifiers that regulate this process remain to be elucidated. Exposed GalNAc residues in the hinge region of Gd−IgA1 can act as neoantigens to stimulate the production of anti−Gd−IgA1 antibodies (139). The interaction between these antibodies and Gd−IgA1 leads to the formation of circulating immune complexes (139, 147). Concurrent intestinal barrier dysfunction may facilitate the translocation of microbial antigens, thereby promoting the formation of additional antigen-specific IgA complexes (11, 15, 144). These immune complexes may evade hepatic clearance by resisting recognition via the asialoglycoprotein receptor pathway, thereby persisting in the circulation (2, 148, 149). Soluble CD89, generated under inflammatory conditions, can bind directly to Gd-IgA1, forming larger and more stable complexes (150). This may further impede its clearance and increase their propensity for vascular wall deposition (148, 149). However, this mechanism is primarily supported by evidence from *in vitro* and animal models. After deposition, Gd-IgA-containing immune complexes can interact with CD89 on circulating myeloid cells, thereby sustaining inflammation and serving as a platform for complement activation in target tissues (151, 152). In HSP, complement system activation converges and amplifies at the level of C3, although the upstream initiation mechanisms may differ from those in IgAN (153–155). This process drives an amplification loop through the formation of C3 convertase, leading to the generation of C5a and the assembly of the membrane attack complex (C5b-9), both of which can mediate cellular injury (155–157). The cascade promotes neutrophil recruitment, oxidative stress, and the release of proinflammatory cytokines such as tumor necrosis factor alpha and interleukin−6 (92–94), ultimately contributing to the development of necrotizing vasculitis (26). Overall, gut microbiota dysbiosis, metabolic disorders, and impaired barrier function may collectively contribute to the production of pathogenic IgA, which in turn contributes to systemic inflammatory responses (2, 8, 158). This inflammatory state may further aggravate immune dysregulation, thereby establishing a potential positive feedback loop that drives the initiation and progression of HSP (26, 27). Notably, most of the described evidence is derived from cross sectional studies, animal models, or *in vitro* experiments. The temporal relationships and causal contributions of these pathways in patients with HSP need to be clarified via prospective studies and intervention trials.

## 4 Emerging therapeutic directions for HSP

**Figure 2:**
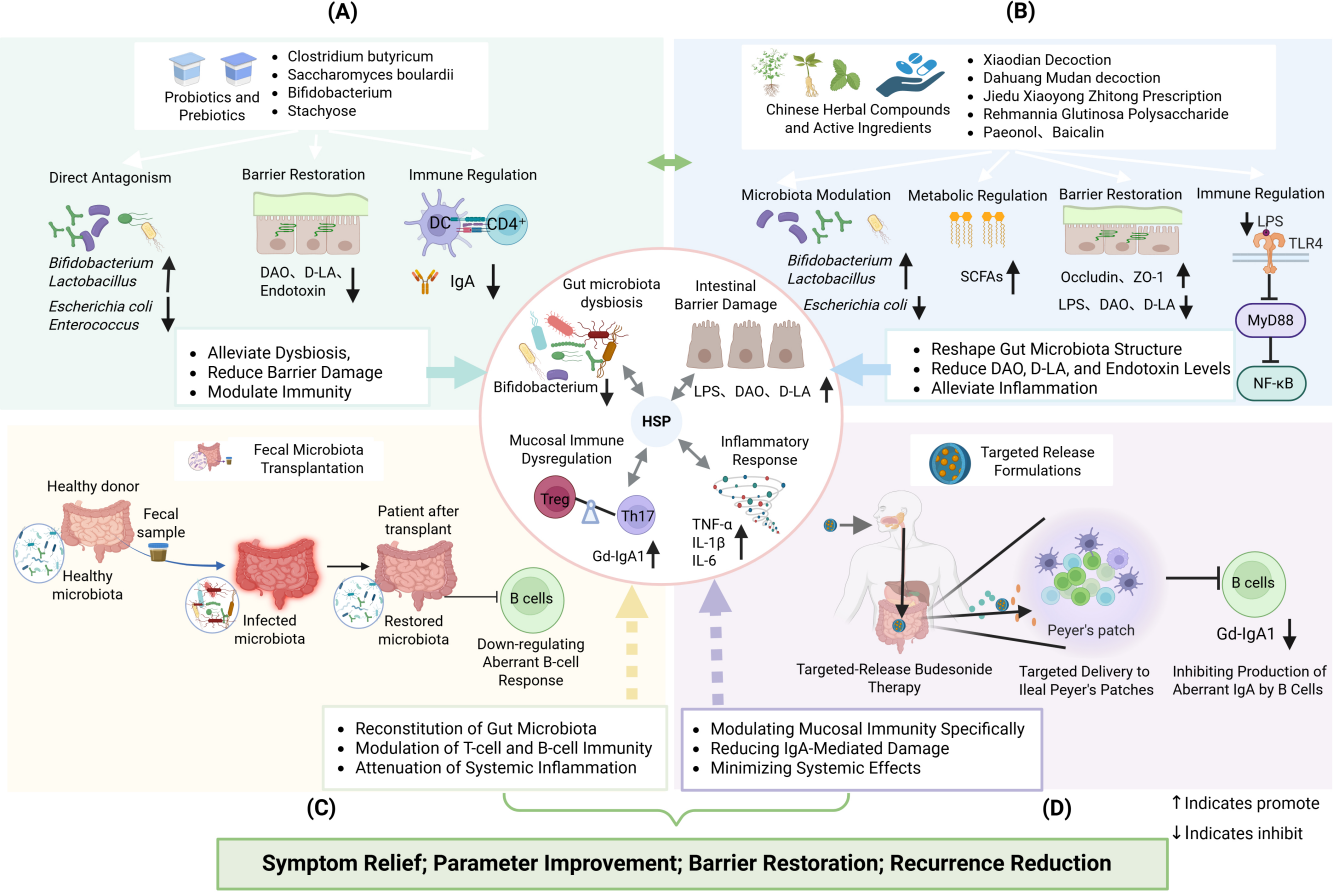
**(A)** Probiotics and prebiotics intervention: competitively inhibits pathogens, restores the intestinal barrier, and modulates immunity; **(B)** Traditional chinese medicine (TCM) compound and active ingredient intervention: Modulates the structure and metabolites of the gut microbiota, restores the intestinal barrier, and regulates immunity; **(C)** Fecal microbiota transplantation (FMT): Reconstructs the gut microbiota, inhibits aberrant mucosal B-cell responses, and reduces pathogenic IgA; **(D)** Targeted-release formulation intervention: precisely delivers the drug to the Peyer's patches in the terminal ileum, inhibits the production of aberrant IgA by mucosal B cells, while the pectin layer exerts a prebiotic effect.

Available evidence suggests that the gut microbiota may be associated with the pathogenesis of HSP. Based on these findings, targeting the gut microbiota has emerged as a promising exploratory therapeutic direction (10, 15, 27) (**Figure 2**).

### 4.1 Probiotic and prebiotic interventions

Probiotics are defined as living microbial organisms that exert beneficial effects on host health via their interaction with the gut microbiota (159, 160). They engage with potential pathogens and commensal bacteria to produce health−promoting metabolites, such as SCFAs, which in turn modulate intestinal mucosal immunity (46, 48). Additionally, probiotics suppress the proliferation of harmful bacteria, strengthen the integrity of the intestinal barrier, and improve the gut microenvironment (70). Adequate probiotic intake can modify gut microbiota composition and mucosal immune status, contributing to reduced intestinal permeability (34). A series of clinical studies has demonstrated that, compared with conventional therapy alone, adjunctive use of probiotics in patients with HSP improves treatment outcomes, corrects intestinal dysbiosis, enhances mucosal barrier function, and alleviates clinical symptoms (161–164) (**Table 2**).

**Table 2:** Summary of key findings from randomized controlled trials on microecological preparations for the treatment of HSP.

Study	HSP type	Intervention	Control	Sample size (n)	Primary outcome measures	Effects on gut microbiota/immune markers
Jiang et al., 2025 (166)	Not specified	*Saccharomyces boulardii* powder (0.25 g/time, 2 times/day, 2 weeks) + control	Montelukast sodium tablets (5 mg/time, once daily, 2 weeks)	108 (54/54)	Total efficacy: 96.30% vs. 85.19% (P<0.05); shortened resolution time of abdominal pain, purpura, arthralgia (all P<0.001)	↑ CD4^+^; ↓ CD8^+^; ↓ IgA; ↓ IL-9, IL-17, TNF-α
Zhang et al., 2022 (162)	Mixed (cutaneous, articular, abdominal)	*Saccharomyces boulardii* powder (1 sachet (0.25 g)/time, 2 times/day, 2 weeks) + control	Montelukast sodium tablets (5 mg/time, once daily, 2 weeks)	90 (45/45)	Total efficacy: 97.78% vs. 86.67% (P<0.05)	↑ CD4^+^; ↓ CD8^+^; ↓ IFN-γ; ↓ IL-4; ↓ Th1/Th2; ↓ IgA
Zhang et al., 2024 (161)	Abdominal type	Bifidobacterium quadruple viable tablets (1.5 g/time, 3 times/day, 1 month) + control	Montelukast sodium tablets (5 mg/time, once daily, 1 month) + conventional treatment	150 (75/75)	Total efficacy: 97.33% vs. 88.00% (P = 0.028)	↑ *Lactobacillus*, *Bifidobacterium*; ↓ *Enterobacter*, *Enterococcus*; ↓ IgA, IgG, IgM; ↓ renal injury markers
Xie et al., 2024 (163)	Abdominal type	Bifidobacterium quadruple viable tablets (3 tablets/time, 3 times/day, 2–4 weeks) + control	Glucocorticoids (dexamethasone → prednisone)	102 (51/51)	Total efficacy: 96.1% vs. 82.4% (P = 0.025); shortened resolution time of abdominal pain, purpura, arthralgia, melena/hematochezia (all P<0.01)	↑ *Bifidobacterium*, *Lactobacillus*; ↓ *Staphylococcus*, *Escherichia coli*; ↓ IgA, IgG, IgM; ↑ CD3^+^, CD4^+^, CD4^+^/CD8^+^; ↓ IL-17
Yao et al., 2022 (167)	Abdominal type	Bifidobacterium triple viable powder (1.0 g/time, 3 times/day, 2 weeks) + control	Conventional treatment	110 (55/55)	Total efficacy: 92.73% vs. 76.36% (P = 0.042); shortened resolution time of rash, abdominal pain, purpura, hematuria, hematochezia (all P<0.001)	↑ serum cholinesterase; ↑ soluble Toll-like receptor 2; ↓ lipid peroxide
Fu et al., 2022 (168)	Abdominal type	Bifidobacterium quadruple viable tablets (3 tablets/time, 3 times/day, 2 weeks) + control	Conventional treatment	102 (51/51)	Total efficacy: 94.12% vs. 80.39% (P = 0.038); shortened resolution time of diarrhea, rash, hematochezia, and hospital stay (P<0.05)	↑ *Lactobacillus*, *Bifidobacterium*; ↓ *Enterococcus*, *Enterobacter*; ↓ DAO, D-lactate, endotoxin; ↓ IgM, IgA, IgG; ↓ Treg, Breg
Liu et al., 2021 (165)	Not specified	*Clostridium butyricum* viable powder (1.0 g/time, 2 times/day, 2 weeks) + control	Conventional treatment	80 (40/40)	Total efficacy: 95.00% vs. 77.50% (P = 0.023); 6-month recurrence: 10.00% vs. 27.50% (P = 0.045)	↑ *Bifidobacterium*; ↓ *Escherichia coli*; ↓ IgA; ↓ complement C3; ↓ urinary protein
Zheng et al., 2019 (164)	Abdominal type	*Clostridium butyricum* combined viable powder (1 g/time, 2 times/day, 4 weeks) + control	Conventional treatment	80 (40/40)	Total efficacy: 95.0% vs. 92.5%, P>0.05; shorter hospital stay (8.89 ± 1.17 d vs. 10.74 ± 1.84 d, P<0.001); reduced recurrence at 3 and 6 months	↓ IgA, IgG, IgM; ↑ CD3^+^, CD4^+^; ↓ CD8^+^, CD19^+^
Tang et al., 2012 (170)	Abdominal type	Stachyose (5 g/day, once daily, 5 days) + control	Bifidobacterium triple viable powder (1 sachet/time, 3 times/day, 5 days) + conventional treatment	58 (29/29)	Cure rate: 65.5% vs. 37.9% (P<0.05); total efficacy: 89.7% vs. 82.8% (P>0.05)	Higher Gram-positive bacilli count on day 10 post-treatment (P<0.001); more stable microbiota

↑ indicates increase/elevation, ↓ indicates decrease/reduction. Conventional treatment refers to antihistamines, antiallergy agents, antiplatelet therapy, fluid resuscitation, bed rest, dietary control, etc. No serious adverse events were reported in any of the studies.

For instance, Liu et al. found that in patients with HSP, the use of *Clostridium butyricum* live bacterial powder via the oral route led to a decrease in *Escherichia coli* levels, an increase in *Bifidobacterium* counts, a higher *Bifidobacterium*-to-*Escherichia coli* ratio, and a reduction in the recurrence rate of HSP (165). Jiang et al. showed that Saccharomyces boulardii powder combined with montelukast sodium tablets significantly increased CD4^+^ levels and the CD4^+^/CD8^+^ ratio, while also reducing serum IgA levels. This combination therapy improved immune function, decreased inflammatory responses, and shortened the duration of skin purpura, abdominal pain, and joint swelling in pediatric HSP (166). Yao et al. found that children with abdominal HSP treated with *Bifidobacterium* triple viable powder exhibited higher serum levels of cholinesterase and soluble Toll-like receptor 2, as well as lower serum lipid peroxide levels, compared with antihistamine treatment alone. Additionally, these children showed improved microcirculation, regulated immune function, reduced cellular damage, and better clinical outcomes (167). Such microecological preparations directly supplement beneficial bacteria, including *Bifidobacterium*, correct microbial imbalance, and restore the Th1/Th2 balance, thereby impeding HSP progression. In a clinical study by Fu et al., administration of *Bifidobacterium* quadruple viable powder led to increased abundances of *Lactobacillus* and *Bifidobacterium*, along with reduced levels of *Enterococcus* and *Enterobacteriaceae*. Additionally, the treatment group exhibited lower concentrations of DAO, D−lactate, and endotoxin compared to the control group, indicating improved integrity of the intestinal mucosal barrier. Moreover, post-treatment levels of IgM, IgA, and IgG were reduced relative to those in the control group, suggesting that adjunctive probiotic therapy can effectively modulate immune responses in HSP (168).

Prebiotics are dietary components that help maintain intestinal microbial homeostasis. They specifically enhance the proliferation and function of beneficial gut microbiota while inhibiting potential pathogens. In this way, they help structure the intestinal microbial community, modulate intestinal immune responses, and defend against foreign antigen invasion (169). Regular intake of foods rich in prebiotics has been demonstrated to positively modulate the gut microbiome and contribute to general health. Common prebiotic substances include inulin, galactooligosaccharides, and fructooligosaccharides. The study by Tang et al. demonstrated that stachyose, acting as a prebiotic, significantly enhances the adjunctive therapeutic effect in pediatric abdominal HSP. In their trial, participants were divided into two groups: the intervention group, which received a combination of *Bifidobacterium* triple viable bacteria and stachyose, and the control group, which was treated with *Bifidobacterium* triple viable bacteria alone. The inclusion of stachyose led to a more substantial and prolonged rise in fecal Gram−positive bacilli. Moreover, the clinical cure rate was markedly higher in the intervention group (65.5%) compared to the control group (37.9%) (170). These findings indicate that prebiotics can promote the proliferation of beneficial gut microbiota, consequently helping to improve the intestinal ecological environment (48, 169).

Accumulating evidence suggests that certain probiotics may act on intestinal epithelial cells. This can occur through direct colonization or via their metabolites. *In vitro* and animal studies have observed that these probiotics promote mucin secretion and enhance physical barrier function (34, 70). Meanwhile, these components can be recognized by intestinal dendritic cells and macrophages, which may subsequently regulate cytokine secretion. As a result, this regulation influences, to some extent, the differentiation trajectory of T cell subsets and the functional activity of B cells (88, 89, 109). In the treatment of pediatric HSP, the addition of prebiotics may theoretically enhance the colonization and proliferation of specific probiotic strains, thereby potentially improving therapeutic outcomes. Current preliminary evidence suggests that, as an adjunctive strategy, probiotics may help shorten disease duration and alleviate clinical symptoms, while prebiotics may exert a synergistic effect by supporting probiotic activity. Nevertheless, the efficacy of probiotics and prebiotics requires further confirmation through high quality studies.

### 4.2 Fecal microbiota transplantation

FMT is a therapeutic procedure that involves the transfer of gut microbiota from the stool of a healthy donor into the gut of a patient, with the aim of restoring the ecological balance of the intestinal microbiome for the treatment of disease (171). This approach has shown promising therapeutic effects in diseases associated with imbalances in intestinal microbial communities and impaired communication between the host and its microbiota. Consequently, it is gaining growing attention as a potential treatment option for immune-related conditions (172). These therapeutic effects are achieved through a multifaceted approach involving the remodeling of the gut microbiota architecture (173), the restoration of the equilibrium among different T lymphocyte subsets (174), the repair of intestinal barrier function to re-establish the normal production of beneficial metabolites such as SCFAs, and the attenuation of systemic inflammatory responses (173, 175). Collectively, these actions are believed to contribute to the re-establishment of intestinal equilibrium and the alleviation of tissue damage (172, 174, 175).

Emerging evidence suggests the promising role of FMT as an adjunctive therapy for Immunoglobulin A nephropathy (IgAN) (176–179). A preliminary clinical study involving 15 patients demonstrated that oral administration of FMT capsules, as an adjunct to conventional antihypertensive therapy (ACEI/ARB), urinary protein levels decreased, peripheral B cell counts were reduced. These changes correlated with alterations in the gut microbiota and metabolites, suggesting that the therapeutic benefit may be mediated through modulation of B−cell immunity (176). Additionally, the potential of this therapeutic approach has been observed in individual cases: following administration of FMT capsules, a patient with refractory IgAN exhibited resolution of proteinuria (from 4.98 g/24h to negative) alongside restoration of gut microbiota diversity (179). In two IgAN patients with severe adverse reactions to immunosuppressive agents, intensive fresh FMT via endoscopic administration resulted in the correction of gut microbiota dysbiosis (characterized by a reduction in Proteobacteria), accompanied by a concurrent decrease in proteinuria (178). Moreover, no serious adverse reactions were observed in any of the aforementioned FMT treatments. In summary, current evidence suggests that FMT may have a favorable safety profile and potential efficacy in reducing proteinuria for the treatment of IgAN, and its mechanism may involve the remodeling of the intestinal microecology and the modulation of systemic immunity (176–179).

While direct clinical evidence supporting the application of FMT in HSP remains insufficient, the two conditions exhibit substantial pathogenic similarities. Both are characterized by disturbances in mucosal immunity, compromised integrity of the intestinal barrier, and alterations in the gut microbial ecosystem. These shared mechanisms offer a theoretical foundation for considering FMT as a potential therapeutic strategy in HSP (5, 15, 38). Future research could consider small-scale clinical trials to preliminarily assess the safety and efficacy of FMT in the treatment of HSP, with particular emphasis on its modulatory effects on the structure of the gut microbiota and serum immune markers in patients.

However, despite its therapeutic potential, the translation of FMT into a routine clinical treatment option remains fraught with substantial challenges. Beyond the necessity of confirming efficacy through large-scale trials, outstanding concerns regarding long-term safety, the potential for transmitting unknown pathogens, and the durability of donor microbiota engraftment remain to be addressed. Further well-designed clinical investigations and randomized controlled trials are required to more thoroughly characterize its safety profile.

Given the predominantly pediatric onset and potentially self-limiting nature of HSP, the application of FMT in this population warrants extreme caution. Rigorous ethical review and long-term safety monitoring are imperative, especially regarding the impact of donor microbiota engraftment on the developing immune system.

### 4.3 Traditional Chinese medicine formulas and bioactive compounds

Traditional Chinese Medicine (TCM) exerts multi-pathway synergistic effects in the treatment of HSP by remodeling intestinal microecological balance, enhancing intestinal mucosal barrier integrity, and consequently modulating immune-inflammatory responses (180–182). A growing body of research indicates that TCM compound formulas and their bioactive components can effectively rectify intestinal microecological disturbances in children with HSP. This intervention contributes to the relief of clinical manifestations and the enhancement of patient outcomes (183–185).

Various TCM formulas have shown therapeutic benefits for managing HSP. A clinical trial by Luo et al. indicated that the combination therapy of Xiaodian Decoction and loratadine led to a notable rise in the intestinal *Lactobacillus* population among HSP patients, in contrast to the group treated with loratadine alone. The combined treatment regimen led to a markedly increased overall response rate (P < 0.05) and showed enhanced effectiveness in alleviating abdominal pain as well as ameliorating microscopic hematuria (183). Li’s research demonstrated that Dahuang Mudan decoction facilitates an alteration of the intestinal microbial composition in pediatric HSP patients, promote the proliferation of *Lactobacillus* and increase lactic acid production, thereby mitigating NF-κB signaling pathway-mediated inflammation in intestinal epithelial cells. It also reduced serum levels of intestinal barrier injury markers such as LPS, DAO, and D-LA, alleviated intestinal mucosal immunoinflammation, and protected intestinal mucosal function. Additionally, it upregulated the production of cytoskeletal components and proteins involved in tight junction formation, which contributed to improved integrity of the intestinal barrier (182). In Gui’s randomized controlled trial on pediatric abdominal HSP, the group administered Jiedu Xiaoyong Zhitong Prescription alongside Western medicine exhibited a significantly elevated marked response rate in contrast to the cohort receiving only Western medicine. After treatment, the experimental group exhibited significant decreases in plasma DAO, endotoxin, and D-LA levels (P < 0.05). While a significant decrease in DAO and D-LA was also observed in the control group, there was no notable change in their plasma endotoxin levels (P > 0.05). The findings indicate that incorporating Jiedu Xiaoyong Zhitong Prescription may yield greater enhancement of intestinal barrier function, potentially through more effective restoration of intestinal filtration capacity (184).

Typically, TCM comprises a range of compounds from various chemical categories, including flavonoids, polysaccharides, and saponins (186, 187). These components exhibit prebiotic properties by fostering the proliferation of advantageous gut microbiota and suppressing the colonization and multiplication of potential pathogens (169). Based on a review of the literature on TCM treatment principles for HSP (188, 189), the most frequently used herbs include Rehmanniae Radix (Rehmannia glutinosa), Moutan Cortex (Paeonia suffruticosa), Scutellariae Radix (Scutellaria baicalensis), and Paeoniae Radix Rubra (Paeonia rubra). The active components of these herbs have been demonstrated to influence the microbial community within the gut, thereby exerting various therapeutic effects (185). Rehmannia polysaccharide is a primary bioactive component of Rehmanniae Radix (187). Research has shown that this compound supports the maintenance of intestinal microbial diversity and promotes the production of SCFAs, thereby contributing to its anti-inflammatory effects (185). Other significant bioactive constituents, including total glycosides derived from Rehmannia leaves and stachyose, have been demonstrated to enhance the growth of beneficial gut microbiota such as *Bifidobacterium*. These effects may contribute to systemic protection through pathways associated with the gut–kidney axis (190, 191). Moutan Cortex’s principal bioactive component, paeonol, regulates the levels of metabolites produced by the gut microbiota, including BAs and SCFAs (192). It enhances the expression of intestinal tight junction proteins such as occludin and ZO-1, decreases intestinal mucosal permeability, and consequently strengthens the intestinal barrier function (192, 193). Furthermore, It modulates adaptive immunity by increasing IL-10 and suppressing IL-17, thereby promoting Treg expansion while inhibiting Th17 differentiation to restore their balance (194). Baicalin, a flavonoid and the principal active component of Scutellariae Radix, ameliorates intestinal inflammation by modulating microbial communities to favor *Butyricimonas* and normalize the Firmicutes-to-Bacteroidetes balance. This elevates butyrate levels, strengthens the gut barrier through upregulation of key junctional proteins and mucin secretion, and enhances anti-inflammatory and antioxidant responses. At the same time, it diminishes pro-inflammatory pathways and oxidative damage (195, 196).

Although direct studies on the effects of these active components of TCM on the gut microbiota in patients with HSP or in corresponding animal models are currently lacking, existing indirect evidence suggests that they may exert certain effects through the modulation of the gut microbiota. This hypothesis warrants validation in future studies through well designed experiments, thereby providing a new perspective for exploring the pathogenesis of HSP and the development of therapeutic strategies.

### 4.4 Targeted-release formulations

In 2021, the FDA granted approval to an oral budesonide formulation designed as a delayed-release capsule that specifically targets the terminal ileum. This therapeutic agent is indicated for decreasing urinary protein excretion among adult patients diagnosed with primary IgAN who face a substantial likelihood of experiencing accelerated renal deterioration (5). Its therapeutic effect is achieved by acting on Peyer’s patches in the distal ileum, leading to a reduction in the levels of Gd-IgA1 (197). The findings from the Phase III NefIgArd study demonstrated that the targeted-release formulation of budesonide is both effective and well-tolerated in adult patients diagnosed with primary IgA nephropathy (198). Furthermore, recent advancements have been made in research on its drug delivery system. A budesonide-loaded yeast microcapsule embedded in a pectin-based gel (NYPs@Gel) can enhance therapeutic efficacy through a dual mechanism: the yeast microcapsules protect the drug and facilitate its targeted delivery to gut-associated lymphoid tissue, thereby modulating mucosal immunity, while the pectin gel coating enables prolonged intestinal retention and sustained release, in addition to acting as a prebiotic to restore microbial balance. Preclinical studies have demonstrated that NYPs@Gel effectively preserves intestinal mucosal barrier function, inhibits aberrant IgA production, minimizes systemic adverse effects, and restores microbial balance in the gut (199). In summary, such local intervention strategies, which effectively modulate the gut–kidney axis, show promising potential for managing conditions with similar pathophysiology, such as HSP.

## 5 Limitations and future directions

Although some progress has been made in elucidating the association between the gut microbiota and HSP, important limitations remain (19, 27). Most study designs in this field are observational and cross-sectional in design, and can only allow the identification of associations without establishing causality (6, 9). Although recent studies on HSP, such as that by Chen et al. (2025), have employed FMT in animal models to directly assess causal direction, their findings suggest that gut microbiota dysbiosis may contribute to the pathogenesis of vasculitis rather than represent a mere consequence of the disease (10). However, a critical unresolved issue remains the directionality of this association, namely, whether gut microbiota dysbiosis functions as an initiating event in HSP pathogenesis or instead arises secondary to systemic inflammation associated with the disease. Although animal experiments support a potential causal role, species-specific differences limit generalizability of these findings to humans and warrants further validation. Although this review focuses primarily on the former hypothesis, the possibility of reverse causality remains equally plausible.

Specific microbial taxa and their metabolites, such as LPS, can activate innate immune pathways and promote Th17 cell differentiation. This provides mechanistic support for the hypothesis that dysbiosis precedes disease onset, as well as preliminary causal evidence from FMT experiments in HSP (10). In contrast, systemic inflammation in active HSP is characterized by elevated levels of proinflammatory cytokines such as IL-6 and TNF-α; these cytokines may directly alter intestinal permeability and reshape the structure of the microbial community (200, 201). Accordingly, we propose a bidirectional “vicious cycle” model, in which an initial trigger (such as infection) induces mild dysbiosis and low-grade inflammation. These changes may further exacerbate intestinal barrier disruption and immune dysregulation, ultimately culminating in clinically manifest HSP. Cross−sectional studies are insufficient for determining directionality in this causal relationship. Longitudinal study designs that track temporal changes in the microbiota and host immune responses are therefore needed. Prospective cohort studies need to be conducted to longitudinally collect fecal samples before disease onset, during acute episodes, and after clinical remission. This would enable tracking of dynamic changes in the microbiota and host responses across the disease course from the acute phase to remission. Such designs would not only help clarify causal direction and temporal sequence, but also facilitate the precise identification of key functional strains and their metabolites through multi−omics analysis (10, 38).

A major limitation of existing clinical studies is the insufficient sample sizes, along with heterogeneity in patient enrollment criteria (such as age, disease stage, and diet) and experimental methodologies; this hinders comparison and integration of results (39). In addition, the translational value of mechanistic findings is constrained by inherent limitations of animal models. These models often fail to fully recapitulate the complexity of human disease; interspecies differences in genetic background, lifestyle, and microbial composition further restrict the clinical applicability of preclinical findings (30).

To address the aforementioned limitations, future research should integrate multiomics approaches. These should include metatranscriptomic profiling to capture gene expression dynamics and metabolomic analyzes to quantify microbial metabolites. Such approaches would facilitate a transition from descriptive compositional analysis (i.e., “who is present?”) to functional characterization (i.e., “what are they doing?”) in the study of HSP pathogenesis. To elucidate the molecular mechanisms underlying therapeutic effects, animal models should be employed to investigate which botanical formulas and their active constituents modulate the gut microbiota in the context of HSP pathogenesis (185, 192). Large-scale, multicenter, long-term randomized controlled trials with standardized protocols are needed to evaluate the efficacy and safety of probiotics, prebiotics, Chinese herbal compounds and their active ingredients, FMT, and targeted-release formulations. These studies should specifically assess their capacity to induce remission, prevent relapse, and preserve renal function (161, 189, 192). Concurrently, a biobank integrating clinical phenotypes, immunological parameters, and metagenomic data should be established. This will facilitate the identification of microbial features that predict treatment response or disease prognosis, thereby providing a foundation for future studies in personalized medicine (39, 41). A causal relationship has not yet been definitively established based on current evidence; however, the bidirectional interaction between the gut microbiota and HSP represents a promising avenue for the development of novel intervention strategies. If the therapeutic effects of microbiota-targeted interventions are validated in future longitudinal cohorts and randomized controlled trials, regulation of the gut microecology may emerge as an adjunctive or potentially central strategy for improving long-term prognosis of patients with HSP (26, 38).

## 6 Conclusion

In summary, accumulating evidence suggests that the gut microbiota may play an important role in the pathogenesis of HSP (27, 29). Several studies have observed that dysbiosis is associated with disruption of the intestinal barrier, increased permeability, and translocation of microbial antigens (20, 39). These events are further associated with innate immune activation and adaptive immune dysregulation, whereby imbalances in the Th17/Treg and Tfh/Tfr cell subsets promote the production of pathogenic Gd−IgA1 (8, 26). The formation and deposition of IgA-containing immune complexes, complement system dysregulation, and classic systemic vasculitic manifestations are recognized features of HSP; however, the precise causal and hierarchical relationships among these processes remain to be elucidated (1, 153).

These findings suggest that the intestinal immune system may play a central role in the pathophysiology of HSP, thereby providing a theoretical basis for the development of novel therapeutic strategies (3, 11, 15). Interventions aimed at modulating microbial balance, such as probiotics, prebiotics, FMT, and specific Chinese herbal formulas, have shown preliminary potential in regulating immune responses and improving clinical outcomes (161, 176, 182, 197). Although establishing causal relationships and integrating these findings into clinical practice remain major challenges, ongoing research into the gut microbiota holds promise for identifying novel therapeutic targets and biomarkers, which may ultimately translate into improved management strategies and long-term outcomes for patients with HSP.
